# Impact of a Remotely Supervised Motor Rehabilitation Program on Maternal Well-Being During the COVID-19 Italian Lockdown

**DOI:** 10.3389/fpsyg.2022.834419

**Published:** 2022-03-07

**Authors:** Moti Zwilling, Alberto Romano, Martina Favetta, Elena Ippolito, Meir Lotan

**Affiliations:** ^1^Department of Economics and Business Administration, Ariel University, Ari’el, Israel; ^2^Department of Health System Management, Ariel University, Ari’el, Israel; ^3^Movement Analysis and Robotics Laboratory, Unit of Neurorehabilitation, Department of Neuroscience, Bambino Gesù Children’s Hospital, Rome, Italy; ^4^Centro AIRETT Ricerca e Innovazione (CARI), Research and Innovation Airett Center, Verona, Italy; ^5^SMART Learning Center, Milan, Italy; ^6^Department of Physiotherapy, Ariel University, Ari’el, Israel; ^7^Israeli Rett Syndrome National Evaluation Team, Sheba Hospital, Ramat-Gan, Israel

**Keywords:** Rett Syndrome, telerehabilitation, parental well-being, exercise therapy, parents, COVID-19, home exercise program

## Abstract

COVID-19 Lockdown was particularly challenging for most mothers of people with intellectual disabilities, including those with Rett syndrome (RTT), leading to feelings of abandonment from healthcare services of their children. Within those days, telerehabilitation has represented a valid alternative to support physical activity and treatment, supporting parents in structuring their children’s daily routine at home. This article aims to describe the well-being level of two groups of mothers of girls and women with RTT who were involved in a home-based remotely supervised motor rehabilitation program, respectively, before and during the COVID-19 Italian lockdown. Forty participants with classic RTT were recruited before the lockdown and randomly assigned to two groups that performed the intervention immediately before (Group 1) and during (Group 2) the lockdown, respectively. The intervention included an individualized daily physical activity program carried out for 12 weeks by participants’ parents and fortnightly supervised throughout Skype contacts to plan, monitor, and accommodate individual activities in the participant’s life at home. The short form Caregivers Well-Being Scale was collected for the mothers in each group 12 weeks before intervention (T1), at intervention initiation (T2), immediately after intervention termination (T3), as well as at 12 weeks after intervention termination (T4). Mothers of participants in the Group 1 showed a stable level of well-being across all four evaluations with a slight improvement during the lockdown, without significant change. Similarly, the well-being level of mothers in the Group 2 showed a statistically significant increase in their well-being between T2 and T3 (during the lockdown) and its reduction to the pre-intervention level between T3 and T4 (after the lockdown). The results suggest that the lockdown did not negatively affect the participants’ mothers’ well-being, leading to its improvement. Moreover, the proposed intervention could have supported the mothers in managing the new daily routine at home, positively affecting maternal well-being.

## Introduction

Rett Syndrome (RTT) is a rare neurological disorder observed mainly in females (Amir et al., 1999, 2000). RTT is characterized by normal birth and apparently normal psychomotor development during the first 6–18 months of life (Epstein, 1995). The disorder’s trademark is the repetitive stereotypical hand movements appearing after the child has entered the typical regression phase of RTT. Additional characteristics at the breakthrough of the disease include autistic-like behavior, panic-like attacks, breathing disturbances, sleeping problems, gait ataxia and apraxia, and acquired microcephaly (Hagberg et al., 1983). After this period of rapid functional deterioration, the disorder progresses relatively stable, although the child with RTT could develop dystonia and musculoskeletal deformities (Sponseller, 2001) as she grows old. Seizures occur in 50–85% (Epstein, 1995; Glaze et al., 2010) of individuals with RTT. Females with RTT typically survive into adulthood, and their estimated life expectancy is 49 years, suggesting the need for life-long support. Due to the complexity of the disorder, RTT is a particularly challenging disorder even for highly trained rehabilitation therapists due to its severity and complexity (Perks et al., 1994).

The challenges presented by the individual with RTT and their expected longevity necessitate constant individual adaptations of intervention in all educational and therapeutic areas. The literature reported that brain development continues after the onset of RTT (Kaufmann et al., 2005). Moreover, the cognitive and communicative abilities of the individual with RTT do not deteriorate over the years. Therefore, setting proper and achievable rehabilitative, educational and communicational goals is vital for the individual with RTT (Cass et al., 2003).

Within the last decade, researchers published new therapeutic strategies for treating each aspect of the disability associated with RTT. These developments include technologically supported strategies for cognitive (Fabio et al., 2020, 2021; Dovigo et al., 2021), communicative (Fabio et al., 2013, 2018b; Stasolla et al., 2014; Vessoyan et al., 2018; Fabio, 2019), and motor (Romano et al., 2020, 2021; Lotan et al., 2021a,b) rehabilitative evaluations and interventions. Technology-supported strategies resulted in adequate support for skills acquisition and improvements in all these fields. The technology-supported strategies available for people with RTT mainly refer to telerehabilitation strategies. Telerehabilitation, also known as net therapy, virtual rehabilitation, mobile rehabilitation, or remote rehabilitation (RR), delivers services over distance *via* technologies addressing therapeutic issues by presenting remote services to individuals with DD (Caprì et al., 2021). Clinically, the term ‘telerehabilitation’ encompasses a range of rehabilitation and habilitation services that include evaluation, assessment, monitoring, prevention, intervention, supervision, education, consultation, and coaching. Technologies used to deliver rehabilitation and habilitation services may incorporate but are not limited to video and audio conferencing, chat messaging, wearable technologies, sensor technologies, patient portals or platforms, mobile health applications, virtual reality, robotics, and therapeutic gaming technologies (Richmond et al., 2017). The benefits of telehealth include improved access to rehabilitation services and specialists, preventing unnecessary delays in care and support (Cason and Cohn, 2014).

Life-long rehabilitation interventions should be performed to maintain individuals with RTT as functional as possible along with their life span, accompanying the person through the disease evolution (George et al., 1988; Lotan and Hanks, 2006; Lotan et al., 2010) and overcoming all the medical and functional impediments mentioned above. Yet, the complexity of the disorder necessitates treatment delivery to the individual with RTT and her family with utmost proficiency and intensity.

Previous findings suggest that an intensive intervention program can enhance the abilities of individuals with RTT in numerous areas, such as learning skills (Elefant, 2001; Koppenhaver et al., 2001a,b; Elefant and Wigram, 2005), new skills (Demeter, 2000; Jacobsen et al., 2001; Leonard et al., 2001), literacy (Koppenhaver et al., 2001a; Hetzroni et al., 2002; Fabio et al., 2013), communication abilities (Sigafoos et al., 2000; Elefant, 2001; Hetzroni et al., 2002; Lindberg, 2006; Wine, 2009; Fabio et al., 2018a), cognitive abilities (Fabio et al., 2011, 2016, 2019, 2020, 2021), manual abilities (Sullivan et al., 1995; Pizzamiglio et al., 2008), osteoporosis (Zysman et al., 2006), functional abilities (Lotan et al., 2004; Downs et al., 2008; Maciques Rodríguez and Lotan, 2011; Lotan et al., 2021a), orthopedic issues (Legrand et al., 1997; McClure et al., 1998; Elefant and Lotan, 2004; Lotan et al., 2004, 2005; Maciques Rodríguez and Lotan, 2011), and sensory issues (Pizzamiglio et al., 2008; Drobnyk et al., 2019). In addition, such programs have been found effective when suggested to individuals with RTT of all ages (Gillberg, 1997; Demeter, 2000; Lindberg, 2006).

As the primary caregivers, the parents and family play a vital role in ensuring the health and well-being of children. The focus of health and developmental services has evolved from a child-centered, traditional “medical” model to a family-centered “developmental” model. Bly suggests that as: “The more involved the family becomes, the more consistent therapeutic management becomes” (Bly, 1999). In this framework, those who coordinate services consider the essential contributions of the family unit and the ability of families to adapt to new challenges. The pediatric healthcare professional must involve family members in all areas of planning, delivery, and evaluation of health and developmental services. Communication between parents and pediatric healthcare professionals should be open, comprehensible, culturally sensitive, and sincere (Ramey and Ramey, 1996). Therefore, the family’s involvement is a fundamental resource. However, in planning the involvement of parents in the therapeutic path of their child with disability, therapists cannot ignore the impact of that decision on the parents’ stress level (Smith et al., 2001). Stressful experiences are considered as person-environment interactions, in which both external stressors and available psychological, socioeconomic, and cultural resources influence the person’s appraisal of the stressor (Lazarus and Folkman, 1984). Stress was found as a multi-dimensional construct that can be operationalized in various ways, often related to having children with developmental disabilities (Crnic et al., 2009). Furthermore, it is known that stress levels negatively influence the well-being of parents of children with developmental disabilities (Cramm and Nieboer, 2011). In general, it can be determined that exposure to prolonged or chronic psychological distress has resulted in adverse health outcomes, ranging from inadequate sleep to negative psychological and physiological well-being (Lee, 2013).

It is known that the process of caring for a child with RTT is, by itself, a difficult task for the family, requiring the activation of psychological, social, and economic resources, and may represent a source of stress for family members (Downs and Leonard, 2016). The risk of increased parental stress level arises from the beginning of the disorder when parents must face the onset of RTT in a child who has shown an initial normal development. Moreover, additional medical comorbidities can arise during childhood, requiring increased attention and constant adaptation of the parents’ caring strategies, all within a framework of uncertainty regarding their daughter’s life expectancy (Mori et al., 2019). Recent articles explored the stress levels of parents of girls with RTT. Authors reported an increased stress level in this population of parents, with mothers showing a higher stress level than fathers (Perry et al., 1992; Pari et al., 2020). Moreover, parental stress level and health-related quality of life were found to correlate with the degree of clinical severity (Sarajlija et al., 2013; Pari et al., 2020). Nevertheless, the stress level appears higher for those parents who had taken care of a girl with RTT for many years and were, on average older, showing a cumulative effect (Mori et al., 2019; Pari et al., 2020). Despite this regression in stress levels, several authors agree that many families with RTT have found functional strategies to cope with the strains of their particular parenting (Perry et al., 1992; Mori et al., 2019). The existing literature agrees in affirming the need for solid support for these parents, which should follow them across their whole parental experience (Perry et al., 1992; Pari et al., 2020). Considering the delicate balance of the need for an intense, long-lasting structured rehabilitative program while maintaining a healthy familial infrastructure, it is necessary to investigate the effects of parental involvement in their daughters’ therapeutic activities on their well-being.

In February 2020, first COVID-19 patients were recognized in Italy. From that moment, the number of affected people started to increase all over the country and abroad and, on March 11th, 2020, the world health organization declared the COVID-19 global pandemic (World Health Organization, 2020). On the same date, the Italian government ordered the first national lockdown closing the majority of the working places, schools, and places of worship and prohibiting all recreational activities. During the lockdown, people have been confined to their houses with the possibility to go out for only a few specific reasons. According to the literature, many Italian parents experienced significant parenting-related exhaustion and well-being levels reduction with increased anxiety, with mothers more severely affected (Cusinato et al., 2020; Marchetti et al., 2020; Bentenuto et al., 2021). Coherent results were found related to mothers of people with disabilities. An Italian study reported increased sleep difficulties and feelings of reduced external support for mothers of children with X-Fragile syndrome. However, their perceived self-efficacy as caregivers did not change during the lockdown (Di Giorgio et al., 2021).

Feelings of abandonment, powerlessness, and fear for their and their children’s health have been reported in numerous studies that have explored the experiences of mothers of both children and adults with intellectual disabilities during the lockdown (Asbury et al., 2021; Embregts et al., 2021; Patel et al., 2021; Rogers et al., 2021). Moreover, in an online survey administered with 527 Italian parents of children with autism spectrum disorder, 93.9% of parents reported that the COVID-19 emergency was a challenging period with difficulties in free time management and structured activities development. The same study reported a low level of specialist support for medical and behavioral needs (Colizzi et al., 2020). Coherently, Bova et al. (2021) analyzed data from interviews conducted with parents of 514 Italian children with neurological disorders who reported that 67.7% of programmed specialist appointments were canceled during the lockdown and about half (49.5%) of children who usually received rehabilitation continued it remotely.

On the other hand, a study from the United Kingdom reported that well-being in families of children with intellectual disabilities measured before and during/immediately after the lockdown did not show any difference. The authors concluded that the general belief of the lockdown’s negative impact on these families could be as straightforward as expected (Bailey et al., 2021). Sporadic similar results were available in other articles that reported benefits for parents due to changes in their daily routines. For these parents, the house confinement represents a possibility to spend more time with their family, strengthening the parent-child relationship and contributing to their well-being (Bentenuto et al., 2021; Embregts et al., 2021; Rogers et al., 2021).

Reports suggest the need for support by most parents of people with intellectual disabilities. Remote rehabilitation has represented a valuable strategy to continue the treatment and support the families in those difficult days (Assenza et al., 2020; Rabanifar and Abdi, 2021). However, patients who have received remote rehabilitation intervention perceived differences in the quality of service and preferred traditional in-person treatment to service delivery *via* remote rehabilitation (Milani et al., 2021). This article aims to describe the well-being level of two groups of mothers of girls and women with RTT who were involved in a home-based remotely supervised motor rehabilitation program, respectively, before and during the COVID-19 Italian lockdown.

## Materials and Method

### Ethical Approval

Declaration of Helsinki principles was followed while conducting this research. The research protocol was approved by Ariel University IRB (AU-HEA-ML-20190326-1) and explained to all participants’ parents, who signed an informed consent form after understanding the protocol and agreeing to participate.

### Participants

Participants were recruited from the Italian Rett syndrome Association database (AIRett). To be included in the current investigation, participants must be genetically diagnosed with classic RTT and reside with their parents. Moreover, participants’ parents must have approved their availability to follow a physical rehabilitation activity program with their daughter for one non-consecutive hour a day, 5 days a week, for 3 months. Candidates for participation were excluded if they presented a neurological or psychomotor developmental deficit comorbidity other than RTT. All candidates were approved to participate by a specialist doctor certified (due to unstable health conditions, e.g., ongoing or recurrent infections, severe gastrointestinal disorders, and drug-resistant epilepsy with multiple daily seizures).

### Study Design

**Figure 1:**
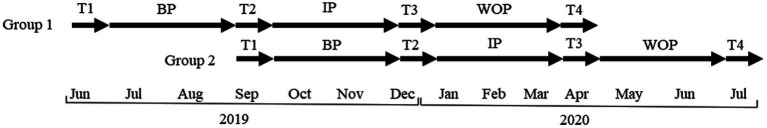
Research timetable. T1–T4 represent the evaluations meeting occurred with 3 months apart. BP, Baseline Phase; IP, Intervention Phase; and WOP, Wash-Out Phase.

A randomized between-groups comparison design was applied. Participants were randomly divided into Group 1 and Group 2. Both groups followed the same A-A-B-A-A protocol. Group 2 started the intervention program 3 months after Group 1. Letter “A” represents the evaluation meetings that occurred 3 months apart. Letter “B” represents the intervention phase. Before the COVID-19 outbreak, when the study was planned, the authors intended to analyze the intervention effects on participants in Group 1 and Group 2 together. However, the Italian COVID-19 lockdown occurred before the beginning of the Group 2 intervention phase occasioning the opportunity to investigate the intervention impact on maternal well-being within the context of the lockdown limitations. Therefore, for the current article, the research protocol planned before the lockdown initiation was not changed due to the COVID-19 outbreak, but the collected data were analyzed for the two groups independently and compared. The research timetable is outlined in Figure 1.

The Participatory Action Research (PAR) method was used. Within the PAR model, participants (in this case, the family members, caregivers, and referral therapists of the person with RTT) are involved throughout the whole research process (Mackenzie et al., 2012). They are asked to participate with the researchers in planning their involvement in the research, identifying problems, and finding solutions to disentangle them through the direct application of research findings in a practical context (Ison, 2014). PAR design is characterized by three recurring stages: inquiry, action, and reflection (Kemmis and McTaggart, 2005). In the current article, the PAR process was developed through the implementation of numerous and continuous cycles of:

- Assessment (of each participant’s therapeutic needs);
- Mutual goal attainment (individualized therapeutic goals were set in mutual agreement with each participant’s parents taking into consideration the participant need and her parents expectation, needs, and availability within the family’s framework);
- Action (the family members implemented the program);
- Reflection (each program was discussed with participant’s family members within the bi-weekly supervised Skype meetings between a trained supervisor and parents); and
- Evaluation (if necessary, the program was modified and re-implemented).

The theoretical framework of PAR allows the participants to actively participate in the research protocol construction they are involved in resulting in greater intervention effectiveness (Newig, 2007). Thus, PAR researchers collaborate with the participants to obtain changes and identify new solutions according to their family’s needs and desires (Ozanne and Saatcioglu, 2008). Even considering the lower level of evidence of PAR design compared to the randomized controlled trial, it best-suited studies that provide a protocol requiring a high level of collaboration between researchers and participants, as is the present investigation (Mackenzie et al., 2012). Moreover, it is the researchers’ intention that the participants’ parents fully understand the process of planning positive physical activity for their daughter (similar to those proposed in the present research) becoming able to develop them even after the research concludes to maintain long-lasting well-being and functional status of the participants with RTT.

### Procedure

All participants’ parents signed an informed consent explaining the research protocol before the research initiation. Each participant was evaluated four times at their house. Evaluation meetings were conducted with 3 months apart from each other (± 1 month, T1–T4). Participants’ mothers’ well-being was assessed during each evaluation meeting.

During the first evaluation meeting (T1), anamnestic information related to participants’ medical situation was collected (e.g., epilepsy, bone density condition, feeding problems, sleep disorders, and other comorbidities typically associated with RTT) together with the extent and type of ongoing physical therapeutic interventions (such as physiotherapy, hippotherapy, hydrotherapy, and others). At the end of this meeting, the researchers compiled a draft of the individualized rehabilitation goals in collaboration with participants’ parents to be pursued in the intervention phase. The objectives set followed the SMART principles to best suit each participant’s potential. Under SMART principles, goals should be specific, measurable, attainable, realistic, and timely (Bovend’Eerdt et al., 2009). No change was made between the first and second evaluation meetings (baseline phase) to the participants’ and their parents’ daily routines.

In the second evaluation meeting (T2), identified treatment goals were re-discussed with participants’ parents, and corrections were applied if needed. At the end of the second (T2) evaluation meeting, an individualized motor activity program was designed for each participant and discussed with the family. Each activity program was developed to pursue the identified intervention goals through easily constructed physical activities that did not require professional competencies to be carried out.

After the second evaluation meeting termination, participants’ parents were asked to conduct the activities of their daughter’s program within her daily routine, for one non-continuous hour a day, 5 days a week, for 3 months. Researchers helped the parents to plan the therapeutic activities development within their weekly routines and habits. After 2 weeks from the delivery of the program, necessary for familiarization with the activities, each family started to participate in remotely conducted supervision meetings with a researcher experienced in the rehabilitation of people with RTT. Supervision meetings occurred fortnightly and lasted for a maximum of 1 h. A videoconference platform (Skype) was used to conduct the supervision meetings. These meetings continued until the end of the intervention phase. The first supervision meeting was mainly dedicated to clarifying any doubts about the practical execution of the activity programs, guiding the parents in the activity development, and modifying them if necessary to meet the families’ needs. The subsequent supervision meetings aimed at supporting the adherence to the program and their execution by answering parents’ questions, adapting the program to emerging needs, solving problems, rearranging the timetable, adapting the proposed exercises, evaluating and sharing the achievement of objectives and, if necessary, setting new goals. Due to the experience of the researchers in RTT, the weekly meetings did not refer only to the program implementation but also to general issues related to RTT, raised by the parents.

After the 3 months of programs implementation, the third evaluation meeting (T3) was conducted. In this meeting, the level of achievement of identified goals was assessed, and the parents’ considerations relating to the intervention phase were collected.

Between the third (T3) and fourth (T4) evaluation meeting (wash-out phase), the remote supervisions were suspended, and parents were informed that they could, at their discretion, continue or interrupt the program. The researcher took this choice to support the continuation of the activities learned, promoting the participants’ physical fitness.

### Measures

#### RTT Severity Level

Rett Assessment Rating Scale (RARS; Fabio et al., 2005) was administered at T1 to assess the participants’ level of RTT clinical severity. This is a 31-item scale aimed to score many specific RTT phenotypic characteristics. Each item is scored on a four-point scale from one to four. Intermediate scores (e.g., score of 2.5) can be attributed to the subject to make the scale more sensitive to the typical variability of RTT. In the theoretical framework of this scale, RTT severity is conceptualized as a continuum between mild deficit (lower score) to severe symptoms (higher score; Vignoli et al., 2010). RARS standardization procedure for the Italian population with RTT was conducted involving a sample of 220 individuals with RTT. Solid psychometric values were proved for this scale (Fabio et al., 2005, 2014; Vignoli et al., 2010; Romano et al., 2020). The results obtained at this scale will be only briefly discussed within the present article. The results obtained from this scale will be presented to provide the reader with a more precise description of the participants’ disease severity.

#### Mothers’ Well-Being

The change in the well-being level across the protocol was evaluated with the short form of the Caregiver Well-Being Scale (CWBS-SF; Tebb et al., 2013). This is a 16-items scale targeted to address areas relevant to caregivers that allow obtaining information related to their well-being. The scale covers basic needs (meeting the biopsychosocial needs to sustain life) and activities of daily living (regarding the implementation of the biopsychosocial needs). A daily need is presented for each item, and the parent is asked to assign a score based on the level he feels he has satisfied that need in the previous 3 months. The score is attributed on a five-point Likert scale from 1 (the need was never or almost never satisfied) to 5 (the need was almost always satisfied). The scores of each item were averaged together for the subsequent analysis. This scale showed an overall internal consistency of 0.83 (Tebb et al., 2013). The CWBS-SF was administered with participants’ mothers only as few fathers participated in all the evaluation sessions.

### Statistical Analyses

The Shapiro-Wilk normality test was used to assess the normality of the data distributions. As most analyzed data sets were not normally distributed, the non-parametric statistic was used to analyze the obtained results. Friedman’s test was run to compare the CWBS-SF scores obtained from the mothers in each group and all together at the four evaluation points. *Post-hoc* analysis with Wilcoxon signed-rank tests was conducted for pairwise comparisons. Mann-Whitney U Test was used to compare the results obtained by Group 1 with those achieved by Group 2 at each time point for all the outcome measures. The Spearman rank correlation coefficient was used to explore the relations between participants’ mothers’ well-being (CWBS-SF score) and participants’ and their mothers’ age and level of participants’ clinical severity at T1 (RARS score). The threshold for significance for the analyses above has been assumed as *α* = 0.05. No correction for multiple comparisons was applied to avoid missing significant results (Armstrong, 2014).

## Results

### Main Descriptive Statistics

Forty-two families were involved in the first evaluation (T1). Two families (4.8%) did not complete the research protocol. One drop-out happened in Group 1 and was due to health problems of the participant’s mother that arose during the baseline period. The other drop-out occurred in Group 2 and concerned a family living in a rural area with negative external involvement by local healthcare professionals who gave them contradicting advice about the proposed program for their child with RTT. Therefore, data of 40 participants with RTT were used for the subsequent analysis. Participants’ and parents’ ages and RTT severity levels measured with RARS were collected in Table 1.

**Table 1:** Descriptive statistics of participants’ and parents’ age, RTT severity level (RARS), and CWBS-SF score.

		Participants’ age	Mothers’ age	RARS score	CWBS-SF			
					T1	T2	T3	T4
All participants (No. 40)	Mean (SD)	15.7(9.7)	50.0(9.8)	67.3(9.8)	3.4(0.6)	3.5(0.6)	3.6(0.7)	3.5(0.8)
	Median	13.3	48.4	67.8	3.3	3.5	3.8	3.5
	Max–Min	40.3–2.8	75.1–29.1	82.5–45.5	4.9–2.2	5–2.4	4.8–2.1	4.8–2
Group 1 (No. 17)	Mean (SD)	16.4(7.9)	53.4(7.1)	66.5(10.7)	3.6(0.6)	3.6(0.6)	3.6(0.7)	3.7(0.7)
	Median	13.3	51.4	68.0	3.6	3.7	3.8	3.8
	Max–Min	38.2–5.4	67.5–39.5	82.5–45.5	3.6(0.6)	3.6(0.6)	3.6(0.7)	3.7(0.7)
Group 2 (No. 23)	Mean (SD)	15.1(11.0)	48.8(10.8)	67.8(9.2)	3.3(0.7)	3.4(0.6)	3.7(0.7)	3.3(0.8)
	Median	13.3	46.7	67.0	3.1	3.4	3.9	3.3
	Max–Min	40.3–2.8	75.1–29.1	82.5–51.5	3.3(0.7)	3.4(0.6)	3.7(0.7)	3.3(0.8)

*SD, Standard deviation; RTT, Rett syndrome; RARS, Rett assessment rating scale; and CWBS-SF, Caregiver well-being scale—short form*.

At the first evaluation meeting (T1), 11 participants were younger than 10 years, 19 were aged between 10 and 20 years, and 10 were older than 20 years. Seven participants attended motor rehabilitative intervention for at least 4 h a week. Twenty-six subjects attended such interventions between 1 and 3 h per week. Five participants were not involved in any motor rehabilitative treatment. All the rehabilitative interventions were suspended during the lockdown. All participants resided at home with their parents. All the participants and their parents were born in Italy. The participants’ and parents’ daily routines and the amount of parents’ working hours varied widely within our sample. In three families, the parents were divorced, and the participants lived with their mother. A weak correlation was found between maternal well-being and both participants’ and mothers’ ages at T1 (*p* = 0.001, rho = 0.491 and *p* = 0.006, rho = 0.429, respectively), T2 (*p* = 0.005, rho = 0.433 and *p* = 0.032, rho = 0.339, respectively), and T4 (*p* < 0.001, rho = 0.530 and *p* = 0.011, rho = 0.398, respectively). No correlation emerged between the maternal well-being level and participants’ RTT severity level.

### Differences Regarding Mothers’ Well-Being

**Figure 2:**
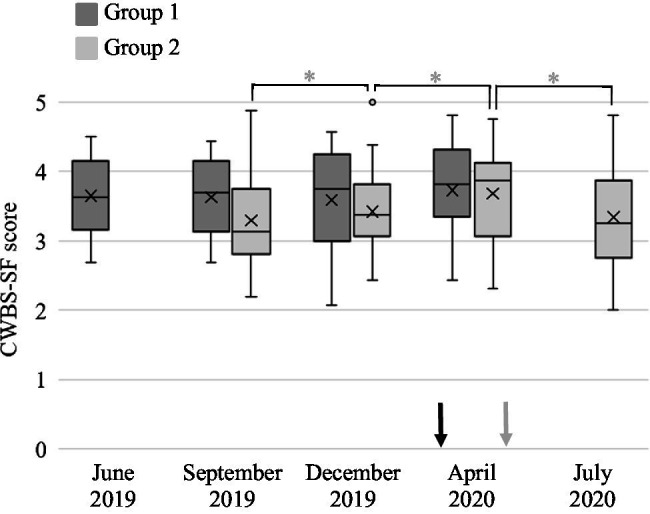
Caregivers’ Well-Being Scale—Short Form (CWBS-SF) score of each group at each evaluation point. The box inferior limits represent first quartile of the distribution, and the upper limits the third quartile (median excluded). The lines across the box show the median score of each group. The crosses inside the boxes mark the mean value of each dataset. The whiskers indicate the minimum and maximum distribution values (outliers excluded). The dots above the upper and below the lower whiskers represent the distribution outliers identified through Tukey’s method (data points that lie above 1.5 times the interquartile range under the first quartile or over the third quartile). In June 2019, Group 1 was evaluated for the first time (T1). In September 2019, the second evaluation was conducted for Group 1 (T2), and the initial assessment occurred for Group 2 (T1). In December 2019, Group 1 was evaluated for the third time (T3) and Group 2 for the second time (T2). In April 2020, Group 1 was assessed for the last time (T4), and the third evaluation was conducted for Group 2 (T3). For Group 2, the last assessment occurred in July 2020 (T4). The black arrow indicates the initiation of the Italian COVID-19 national lockdown, and the grey arrow indicates its end for a total of 2 months. ^*^p ≤ 0.05.

CWBS-SF was assessed to investigate the impact of program implementation on participants’ mothers’ well-being. Descriptive statistics of CWBS-SF are collected in Table 1. The variation of maternal well-being within the current project differed consistently between Group 1 and Group 2 (see Figure 2).

**Figure 3:**
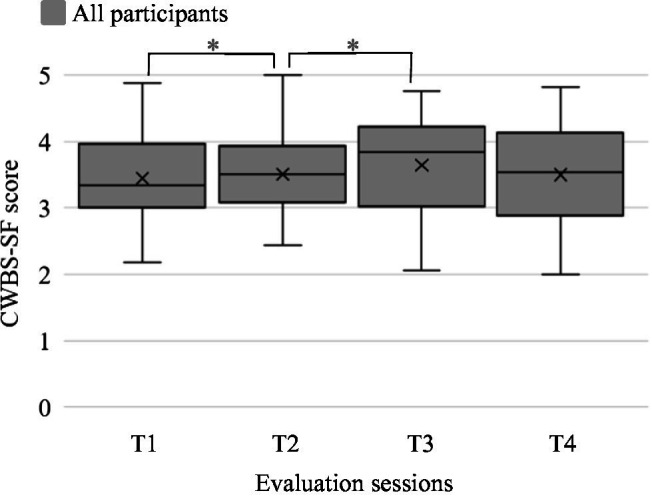
Caregivers’ Well-Being Scale—Short Form (CWBS-SF) score of all participants together at each evaluation point. The box inferior limits represent first quartile of the distribution, and the upper limits the third quartile (median excluded). The lines across the box show the median score of each group. The crosses inside the boxes mark the mean value of each dataset. The whiskers indicate the minimum and maximum distribution values. ^*^p ≤ 0.05.

However, when analyzed with the Mann-Whitney U Test, no statistical difference emerged between mothers in Group 1 and Group 2. Mothers in the Group 1 showed a stable level of well-being across all four evaluations with a slight improvement of median well-being level between T1 and T4. These changes were not statistically significant at any conducted analysis. Conversely, the well-being level of mothers in the Group 2 showed a significant change across the four evaluations at the Friedman test (*p* = 0.012). Significant increases in mothers’ well-being were found between T1 and T2 and between T2 and T3 (*p* = 0.002; *p* = 0.013, respectively), but a statistically significant reduction between T3 and T4 (*p* = 0.031) was found. Looking at participants’ mothers altogether (see Figure 3), the Friedman test identified no significant score change among the four evaluation meeting. However, the maternal well-being showed a significant increment between T1 and T2 (*p* = 0.018) and between T2 and T3 (*p* = 0.050) and then slightly reduced at T4, but without reaching the statistical significance with any of the other evaluations.

Moreover, on average, the CWBS-SF score in our group remained within the range described as acceptable for families without children with disabilities (average: 3.6—range: 2.7–4.9; Tebb et al., 2013). No correlation emerged between the mothers’ well-being level and the other outcome measures.

## Discussion

This article described the well-being of mothers of girls with RTT and how it was affected by their enrollment in their daughter’s rehabilitation program during the COVID-19 lockdown in Italy. A motor activity program was given to each involved family to be carried out at home and was fortnightly remotely supervised through video calls. Parental well-being was assessed before and after treatment. However, on March 9th, the Italian government established the first national lockdown to face the COVID-19 outbreak. This has led to the interruption of most working and recreational activities and to the obligation for all Italian citizens to stay at home and go out only if strictly necessary. In this phase, rehabilitation and assistance facilities for people with disabilities and schools were also closed. The lockdown and the related restrictions continued until May 4th, when most work activities were resumed, but without the reopening of shops, restaurants, cafes, and places of worship that occurred on May 18th. On the same day, some, but not all, rehabilitation activities for people with disabilities were also resumed. However, the limitations to some recreational activities, such as cinemas and theaters, and the attendance of summer camps for children continued until June 11th, and school attendance did not start again until September 2020. The restriction progression caused the parents to spend 2 months at home with their families without working activities. Subsequently, after the recovery of the working activity (May 4th), the parents’ situation has changed. They were asked to go back to work within a context of social distancing, reduced availability of recreational activity, fear of contagion, and with their child at home from school and rehabilitation facilities.

In this study, two groups of people with RTT and their families followed the same research protocol starting with 3 months apart. For this reason, the lockdown occurred in a different phase of the protocol for the two groups. For Group 1, the restrictions began within the wash-out phase while, for Group 2, the lockdown started during the intervention phase (see Figure 1). The mothers’ well-being scores across the four evaluation meetings differed for the two groups. The mothers in Group 1, after stable well-being scores in the first three evaluation meeting, showed a slight increase at T4 (during the lockdown). Similarly, the well-being of mothers in the Group 2 increased at baseline and, more markedly, during the intervention phase, before going back to the pre-intervention level at T4. These results suggested that the lockdown did not negatively affect the mothers’ well-being level but increased it. This effect can be explained by the fact that the lockdown situation allowed the mothers to spend more time with their families in the absence of working activities. These results are correlated to the findings of Bailey et al. (2021) and with sporadic results from other studies (Bentenuto et al., 2021; Embregts et al., 2021; Rogers et al., 2021).

Nevertheless, within the lockdown, mothers in Group 2 showed a more markedly improved well-being than those in Group 1. These can be explained by the presence of the remote supervision meeting that occurred during the lockdown for Group 2 but before it for Group 1. The literature reports that parents of children with intellectual disabilities suffered the reduction in healthcare professional support they received through the lockdown (Asbury et al., 2021; Embregts et al., 2021; Patel et al., 2021; Rogers et al., 2021). This did not happen in the case of mothers in group 2 who received an organized program to implement and constant follow-up talks where they could unload their fears and concerns.

Furthermore, the positive correlation between mothers’ and participants’ age and maternal well-being suggests that older mothers have higher well-being levels than younger ones contrasting with published literature (Perry et al., 1992; Pari et al., 2020). The fact that both articles investigated parental stress while our study focused on maternal well-being could explain this difference. However, both Perry et al. (1992) and Pari et al. (2020) suggested that families with RTT frequently find suitable strategies to cope with the strains related to their parental role. In this light, our findings agree with the researches mentioned above, as shown by the average well-being level of parents in our groups that remained in the range reported for parents without children with disabilities (Tebb et al., 2013). However, these results may also relate to the fact that all participating families remained protected from the COVID-19 contagion and were financially well established, thereby reducing the burden associated with the pandemic.

The current investigation presents some limitations. First, only one measure of maternal well-being was used. For more solid results, more evaluation tools should be used to investigate more dimensions of maternal well-being and stress level in the future. Moreover, the amount of time each parent spent in their daughter’s program was not collected, but we asked them never to exceed 1 h of treatment per day for 5 days a week. Furthermore, a relatively small sample was enrolled in this study, challenging the external validity of the obtained results. Additionally, each group’s participants’ data were analyzed together, preventing further fine-grained analyses. As a correlation between the participants’ ages and their mothers’ well-being level was identified, the age effect may have affected the whole group’s well-being outcome. Therefore, future studies are needed to evaluate the impact of the participants’ and parents’ ages on the well-being of parents involved in activity programs, such as the one presented. Plus, the maternal working status changes due to the lockdown were not assessed within the present study limiting the validity of the discussion of the results. However, most workplaces were closed, and people were forbidden to leave their houses (except for a few reasons) within those months in Italy. Therefore, the authors reasonably assumed that, on average, the participants’ mothers’ working hours were reduced during the lockdown. Similarly, it would have been interesting to analyze the amount of social support the mother received in the different phases of the present study and their impact on maternal well-being. Finally, only mothers’ well-being was analyzed due to fathers’ missing data. Future research should investigate these variables individually for each parent to understand the differences between the impact of the treatment on both mothers and fathers.

## Conclusion

The results obtained at the CWBS-SF hint that the availability of an activity program and the conducted remote supervision calls positively affected maternal well-being in a challenging period, such as the COVID-19 lockdown. Moreover, as the researchers were highly familiarized with RTT, their bi-weekly calls enabled the parents to consult on other issues which were not directly connected with the motor elements of the program. It is the authors’ opinion that the strategies and suggestions given to parents within the current project supported the daily caring of their daughters, supporting the maternal well-being in accordance with existing literature, not specifically related to RTT (Singer et al., 2007; Todd et al., 2010).

## Data Availability Statement

The original contributions presented in the study are included in the article/supplementary files, and further inquiries can be directed to the corresponding author.

## Ethics Statement

The studies involving human participants were reviewed and approved by the Ariel University Institutional Review Boards, Ariel University, Ari’el, Israel. Written informed consent to participate in this study was provided by the participants’ legal guardian/next of kin.

## Author Contributions

ML obtained the funds. MZ and ML coordinated the project. ML and AR conducted participants’ evaluations. AR organized the participants’ evaluations and carried out all remote supervision meetings. EI and MF collected the data that MZ and AR analyzed. MZ, ML, and AR wrote the article. All authors have read the article and suggested improvements and changes until agreement was reached.

## Funding

The International Rett Syndrome Foundation funded the described project within the HeART grant no. 3610.

## Conflict of Interest

The authors declare that the research was conducted in the absence of any commercial or financial relationships that could be construed as a potential conflict of interest.

## Publisher’s Note

All claims expressed in this article are solely those of the authors and do not necessarily represent those of their affiliated organizations, or those of the publisher, the editors and the reviewers. Any product that may be evaluated in this article, or claim that may be made by its manufacturer, is not guaranteed or endorsed by the publisher.
